# Six facial prosodic expressions caregivers similarly display to infants and dogs

**DOI:** 10.1038/s41598-022-26981-7

**Published:** 2023-01-17

**Authors:** Anna Gergely, Édua Koós-Hutás, Lőrinc András Filep, Anna Kis, József Topál

**Affiliations:** 1 Institute of Cognitive Neuroscience and Psychology, Research Centre for Natural Sciences, 2 Magyar Tudósok Krt., Budapest, Hungary; 2 Doctoral School of Psychology, Eötvös Loránd University, 46 Izabella U., Budapest, Hungary

**Keywords:** Human behaviour, Animal behaviour

## Abstract

Parents tend to use a specific communication style, including specific facial expressions, when speaking to their preverbal infants which has important implications for children’s healthy development. In the present study, we investigated these facial prosodic features of caregivers with a novel method that compares infant-, dog- and adult-directed communication. We identified three novel facial displays in addition to the already described three facial expressions (i.e. the ‘*prosodic faces*’) that mothers and fathers are typically displaying when interacting with their 1–18 month-old infants and family dogs, but not when interacting with another adult. The so-called *Special Happy* expression proved to be the most frequent face type during infant- and dog-directed communication which always includes a Duchenne marker to convey an honest and intense happy emotion of the speaker. These results suggest that the ‘*prosodic faces*’ play an important role in both adult-infant and human–dog interactions and fulfil specific functions: to call and maintain the partner’s attention, to foster emotionally positive interactions, and to strengthen social bonds. Our study highlights the relevance of future comparative studies on facial prosody and its potential contribution to healthy emotional and cognitive development of infants.

## Introduction

### Importance of infant-directed prosody

When interacting with infants, adults adjust their communication style to the needs and abilities of the baby and this adjustment process can be characterized by specific intonation, uttering, body, and facial movements (for a review see^1^). Caregivers use this specific multimodal infant-directed (ID) prosody for multiple although not mutually exclusive purposes. These functions can be divided into two main categories: (i) cognitive functions (language tutoring—e.g.^2^; conveying information about the talker’s intentions and identity—e.g.^3,4^) and (ii) affective functions (getting and maintaining the infant’s attention—e.g.^5,6^; enhancing positive interactions with the infant—e.g.^7^; strengthening bonding between the interaction partners—e.g.^8^; expressing emotions—e.g.^9^; supporting emotional synchrony between interactants—e.g.^10^).

Several studies provided evidence that effective prosody, which can accomplish the aforementioned cognitive and affective functions, contributes greatly to young children’s cognitive and emotional development. Less pronounced ID prosody is one of the typical symptoms of postpartum depression (PPD), and it is a potential risk factor for poor infant mental health (e.g.^11^). In line with this, children of mothers with PPD are more vulnerable to developing mental disorders later in life (for a review see^12^). It has been also shown that less exaggerated and less salient prosody of depressed mothers leads to failure in simple associative tasks in 4-month-old infants^13^. Moreover, infants of healthy mothers, who have been trained how to produce typical ID prosody, were more successful in linguistic tasks than their contemporaries whose mothers did not go through such training^14^.

While ID prosodic features are clearly multimodal exchanges, research has so far mainly focused on the acoustic components, and less is known about the visual domain, especially the specific facial expressions, that adults display when interacting face-to-face with infants. Without detailed investigations of ID facial prosody, we have limited knowledge of how and to what extent the visual domain contributes to healthy development of infants.

### Infant-directed facial prosody

Chong and co-workers’ paper^15^ is one of the few studies focusing on ‘visual prosody’ in mother-infant interactions. The authors in this study have identified and described unique muscle movements of mothers in the form of three distinct facial expressions, each conveying a mixture of positive emotions like joy, happiness, surprise, excitement, and interest. These facial expressions can also be observed in adult-directed (AD) communication, yet differ from them in certain characteristics and/or intensity^16^. One typical infant-directed (ID) facial expression is the so-called ‘mock surprise’ which was described by Stern^17^. It is characterized by exaggerated brow raise, open-stretched mouth with a hint of a smile, and accompanied frequently with ‘Ooooo’ vocalizations^15^. Another ID-specific facial display is the ‘fish face’^17^ characterized by puckered lips with a moderate smile and brow raise. Authors suggested a soothing-comforting function of this facial expression^15^. Third, and the most frequent one is the so-called *Special Happy* expression, characterized by intense lip corner pull, cheek raise, and slightly open mouth^15^. It’s important to note, that according to Chong et al.^15^ all three ID faces contain some type of smile, and the *Special Happy* is basically an intense Duchenne smile (in which zygomatic activity is combined with contraction of the orbicularis oculi—see^18^). Since all ID-specific expressions are typically displayed both by English and Chinese mothers, Chong et al.^15^ concluded that these are probably universal (culture independent) and highlighted the importance of further investigations of the facial expressions that potentially convey an extra layer of meaning in infant-directed interactions.

Another interesting aspect of ID communication is the effect of the speaker’s gender on facial prosody. Although compared with mothers, fathers are underrepresented in the literature on adult-to-infant communication, some studies have provided evidence of gender-based differences in the use of facial prosody. It has been shown, for example, that distinct smile types (i.e. basic- or Duchenne smiles) can occur when observing mother-infant as opposed to father-infant dyads during natural playing sessions^19^. There is also evidence that parents have different interactive styles and father-infant interactions are more playful and arousing with sudden peaks in valence and different temporal patterns of happy emotion when compared with mother-infant interactions^10,20^. Because of differences in maternal and paternal face-to-face interactions with infants and due to the importance of fathers in healthy development of infants^21^, gender differences in facial prosody call for a more detailed investigation.

In addition to the gender-specific features, context-related (i.e. situation specific) differences in ID facial prosody is also worthy of mention. Facial prosody is strongly affected by the situation (e.g. naturalistic versus laboratory observations), as well as the linguistic and affective content of the interaction (see, e.g.^22^ for a review). Mothers smile more when they sing than when they talk to their infants^23^. Mothers tend to produce more Duchenne smiles during an object play situation (in which they were speaking freely) as opposed to book reading or singing (with fixed sentences), while in fathers, book reading elicits more Duchenne smiles than other play situations^19^. Chong and co-workers^15^ recorded mothers’ ID and AD facial expressions only during a free speech situation (i.e. without any instruction or restriction to the linguistic content) in which they were instructed to talk about six topics for at least 15 s. These topics were aimed to evoke different emotional content and expressions, like “tell a story about the first diaper change to your baby” or “tell a story about your feelings toward your baby” etc. Importantly, however, the authors did not analyse whether or not ID facial prosody of the mothers differed in terms of topics, thus the situation specific features of ID specific facial expressions (i.e. *Special Happy*, *Mock Surprise* and *Fish Face*) remained unknown.

### A comparative framework for studying facial prosody

It is known, however, that ID prosody may not be uniquely used toward pre-verbal humans; similar speech forms have also been described toward pet companions such as dogs^24,25^, parrots^26^ and cats^2,27^. This potentially suggests that ID-like communication forms are widespread towards non-speaking con- and heterospecific partners. Based on the increasingly important role of dogs in interspecific comparative investigations^28,29^ here we propose that human–dog interaction in general, and dog-directed (DD) prosody in more particular, is a feasible option for studying the role that facial prosody plays in ID communication.

There is growing evidence supporting the viability of this approach. Family dogs show infant-like sensitivity both to the acoustic and to the facial features of ID prosody, like heightened pitch and happy facial expression^30–33^. This suggests that the attention getting function of ID and DD prosody might work similarly. Dogs also show similar patterns of attachment behaviour toward their caregiver as infants toward their mothers^34^. It is reasonable to assume, therefore, that DD prosody has an important role in enhancing the human–dog bond. Similarly to ID communication, gender- and situation specific features are also present in dog–human interactions^25,35^, which also make this comparative approach feasible and productive.

Besides these similarities, there are important differences between human infants’ and family dogs’ cognitive abilities and emotional processing. Dogs are non-conspecific social partners with different anatomy and the capacity to display facial expressions as compared to humans^36,37^. There is also evidence that they process human faces differently than humans do (e.g.^38,39^). These differences can have a significant effect on the reciprocity of emotional expressions, a key function of prosody, and suggest that mirroring events (i.e. when mothers and infants display similar facial expression one after another^40,41^) are probably rare or completely missing from an interaction with a dog. Last but not least, dogs, compared to infants, have a limited understanding of word meaning^42,43^ and intentions of others^44^, and are not capable of forming higher-order representations^45^. In line with these findings, Burnham and co-workers^2^ showed that hyperarticulation of vowels is missing from DD as opposed to ID communication. They suggest that hyperarticulation has a didactic function (language tutoring) which is presented only when we are talking to prospective speakers (i.e. infants) but not when speaking to non-human animals (see also^25,26^). Thus it is reasonable to assume that unlike in ID communication, cognitive functions of prosody have a minor, if any, role in DD communication. In sum, these similarities and differences make DD prosody a feasible model to study the species specific features as well as separate cognitive and affective functional features of facial prosody.

### Aims and hypothesis

In light of the above research findings, the present paper aims to provide a detailed analysis of maternal and paternal facial prosodic characteristics of ID communication in comparison to AD and DD communication during natural face-to-face interactions. In addition, we were also curious about the gender- and situation specific features of ID, DD, and AD facial prosody. To do so, we recorded the speaker’s facial prosody during two free-speech situations with different object-related playing tasks and one-fixed speech situation (see Methods for details). Based on previous studies, these situations have the potential to evoke typical ID, DD and AD communication style^25^ and are also feasible to study gender-related features of facial prosody toward infants^19,22,23^. We coded and analysed the occurrence (i.e. frequency) and intensity of facial expressions displayed during ID, DD and AD communication including those three ones that have been described in earlier studies (i.e. *Special Happy*, *Mock Surprise,* and *Fish Face*^15,17^). The effects of the speaker’s gender, as well as the situation specific details of the facial expressions were also investigated by comparing the three situations within and between female and male speakers.

The questions we set out to address were the following:

(i) Whether speakers display similar facial prosody toward infants, dogs, and adults? Based on the aforementioned similarities and differences between communication directed towards infants’ and dogs’^2,24–26^ we hypothesised an increased, but not identical, presence of prosodic facial expressions in both infant- and dog- directed (as compared to adult-directed) communication.

(ii) Whether facial prosody toward infants, dogs, and adults differ between female and male speakers and/or situations? Since previous studies suggested that happy facial expressions, including different smile types, differ between situations and this difference was affected by the speaker’s gender^19^, we hypothesized interaction effects between gender and situation in terms of types (and frequency) of facial prosodic features. More specifically, more frequent and intense *Special Happy* expression, that includes a Duchenne smile, are expected during object-related situations as opposed to the fixed-speech situation in female speakers, and an opposite tendency is predicted in male speakers^19^. We also hypothesized that the occurrence and intensity of the facial prosodic features will be different in the more naturalistic free speech and less naturalistic fixed speech situations in both genders^22^.

## Results

### Categorization of facial expressions: the ‘prosodic faces’

We have found that 65.2% of the pictures fit into the three predetermined categories of facial expressions (*Fish Face, Mock Surprise, Special Happy—*see^15,17^). But in contrast to former studies, we also provided a detailed description of facial movements (including relevant AUs, activation percentages, and intensities) using the Facial Action Coding System (FACS) (see Table 1):

**Table 1:** Intensity of the facial muscle movements presented as separated Action Units (AUs, mean ± SE) and percentage (%) of AU activation (i.e. intensity score differed from 0) in the six ‘prosodic faces’.

	Special happy	Mock surprise	Fish face	Mock surprise brow	Mock surprise mouth	Mock surprise + special happy
N (1056 in total)	543	114	32	180	72	115
Brow (AU1 + 2)	0.09 ± 0.41 12.38%	**3.07 ± 1.73** **93.86%**	**0.94 ± 1.7** **56.25%**	**3.03 ± 1.35** **100%**	0.58 ± 0.9 44.44%	**3.95 ± 1.27** **100%**
Eyelid (AU5-AU44)	**−0.83 ± 1.38** **52.3%**	**1.78 ± 1.71** **69.64%**	0.5 ± 1.29 42.86%	**1.62 ± 1.59** **63.84%**	0.5 ± 1.22 35.71%	**1.73 ± 1.95** **91.96%**
Cheek (AU6)	**4.35 ± 0.84** **100%**	0.09 ± 0.39 6.14%	0.61 ± 0.99 35.48%	0.12 ± 0.44 8.33%	0.04 ± 0.26 2.77%	**4.02 ± 1.16** **100%**
Lip corner (AU12)	**4.27 ± 0.86** **100%**	0.79 ± 1.03 48.25%	0.06 ± 0.35 3.13%	**0.94 ± 1.06** **55%**	0.5 ± 0.95 27.78%	**3.93 ± 1.06** **99.13%**
Lip pucker (AU18)	0.05 ± 0.27 3.5%	0.9 ± 1.57 35.09%	**3.25 ± 1.08** **100%**	0.55 ± 1.05 28.89%	0.42 ± 1.11 16.67%	0.35 ± 0.91 16.52%
Lips part (AU25)	**4.32 ± 1.11** **98.12%**	**4.42 ± 1.25** **97.35%**	**2.97 ± 1.62** **93.55%**	**3.15 ± 1.84** **86.36%**	**4.81 ± 0.62** **100%**	**4.3 ± 1.01** **100%**
Mouth opening-jaw drop (AU26-27)	0.32 ± 0.63 33.77%	**1.96 ± 1.18** **92%**	**0.81 ± 0.65** **79.31%**	0.42 ± 0.58 47.9%	**2.37 ± 1.09** **100%**	**1.31 ± 1.55** **62.83%**

Bold highlight represents relevant AUs (i.e. which was active in more than 50% of the cases).

### Fish face—FF

**Figure 1:**
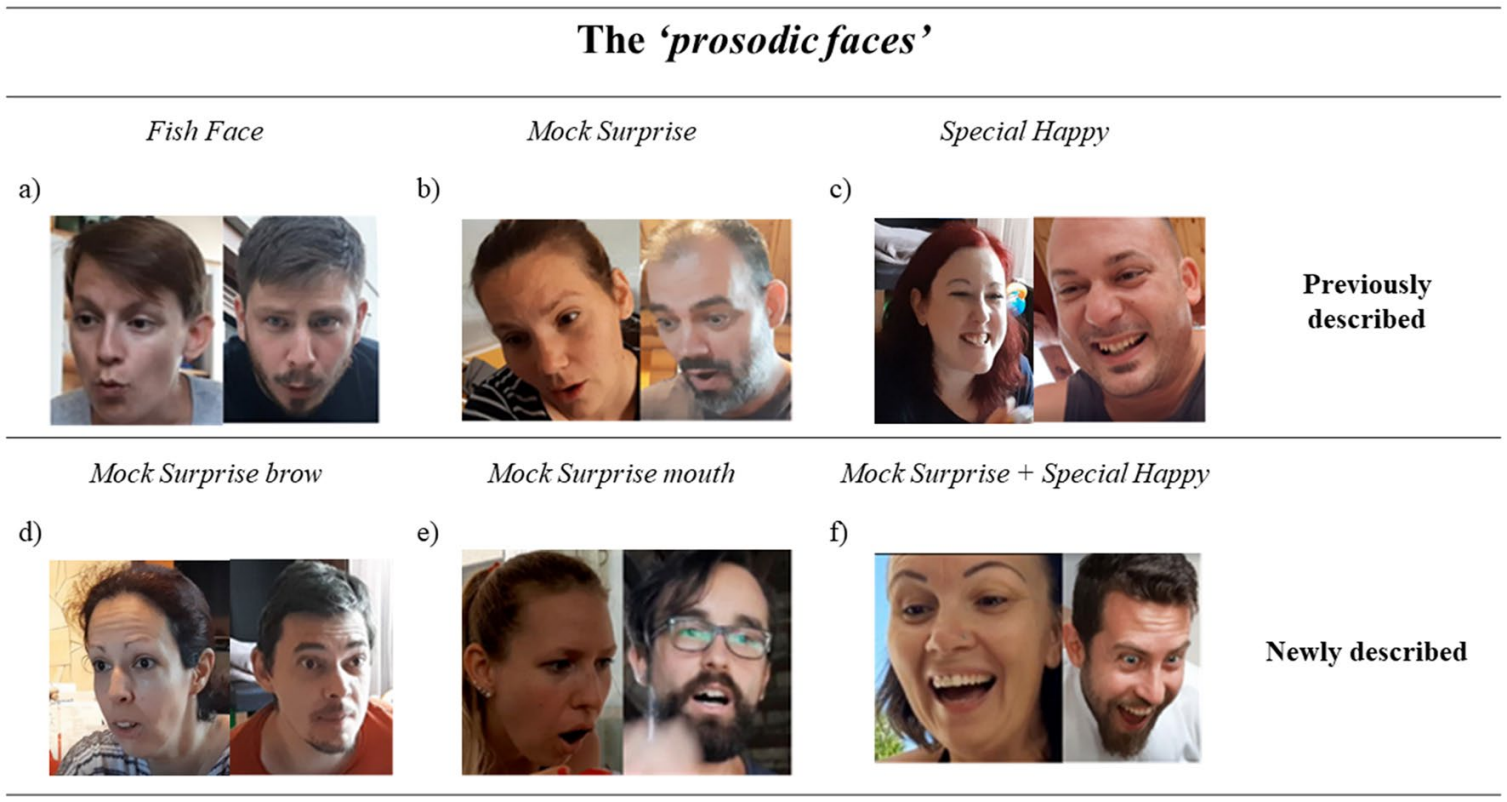
The *‘prosodic faces’*: three facial displays from Stern^17^ and Chong et al.^15^ and three additional faces that we have identified in the present study.

Similarly to previous studies^15,17^ we found that this facial expression can be characterized by the crinkled lips (activation of AU18, AU22) and the slightly open mouth create (AU25, see Fig. 1a).

### Mock surprise—MS

Similarly to previous studies^15,17^ we found that this facial expression can be characterized by the simultaneous appearance of the raised eyebrows (AU1 + 2) and wide-open eyes (AU5) and mouth (AU26, AU27, see Fig. 1b).

*Special Happy- SH*: In line with previous studies^15,17^ we found that this facial expression can be characterized by a large lip corner pull (AU12) accompanied by a definite cheek raise (the Duchenne marker, corresponding to AU 6) and slightly opened mouth (AU25, Fig. 1c).

Importantly, however, we have also successfully identified three new facial expressions as a result of FACS coding (see Table 1):

### Mock surprise brow (MS(brow))

The *MS(brow)* lacks great movements of the mouth, but a pronounced raise of the eyebrows are present similarly to *MS* (AU1 + 2, Fig. 1d).

### Mock surprise mouth (MS(mouth))

The typical feature belonging to this expression is the wide-open mouth (AU26, AU27) occurring in *MS*, but the upper side of the face stays rather neutral, and no pronounced eyebrow movements are observable (Fig. 1e).

### Mock surprise + special happy (MSSH)

This label refers to a particular combination of the *MS* and *SH* expressions characterized by eyebrow raise (AU1 + 2) as well as cheek raise (AU6) and an intensive lip corner pull (AU12, Fig. 1f).

Although all of these ‘novel’ expressions are mixtures or subtypes of the above-mentioned predetermined facial expressions (*FF; MS; SH*), these faces have characteristics clearly distinguishable from those of described by Chong et al.^15^ and Stern^17^. Therefore we decided to consider and analyse these six facial expressions (hereinafter called ‘*prosodic faces*’) separately.

### Frequency analysis

As a next step we analysed the effects of partner, situation, speaker’s gender and the type of the expression on the frequencies of the ‘*prosodic faces*’. After removing the non-significant interaction terms, the final model revealed a significant three-way interaction effect of Partner × Situation × Face Type (F_34,2208_ = 1.78, p = 0.004) and two-way interaction effect of Gender × Face Type (F_5,2208_ = 3.48, p = 0.006, for details see Supplementary Material 1.1 and Table S1a-c).

**Figure 2:**
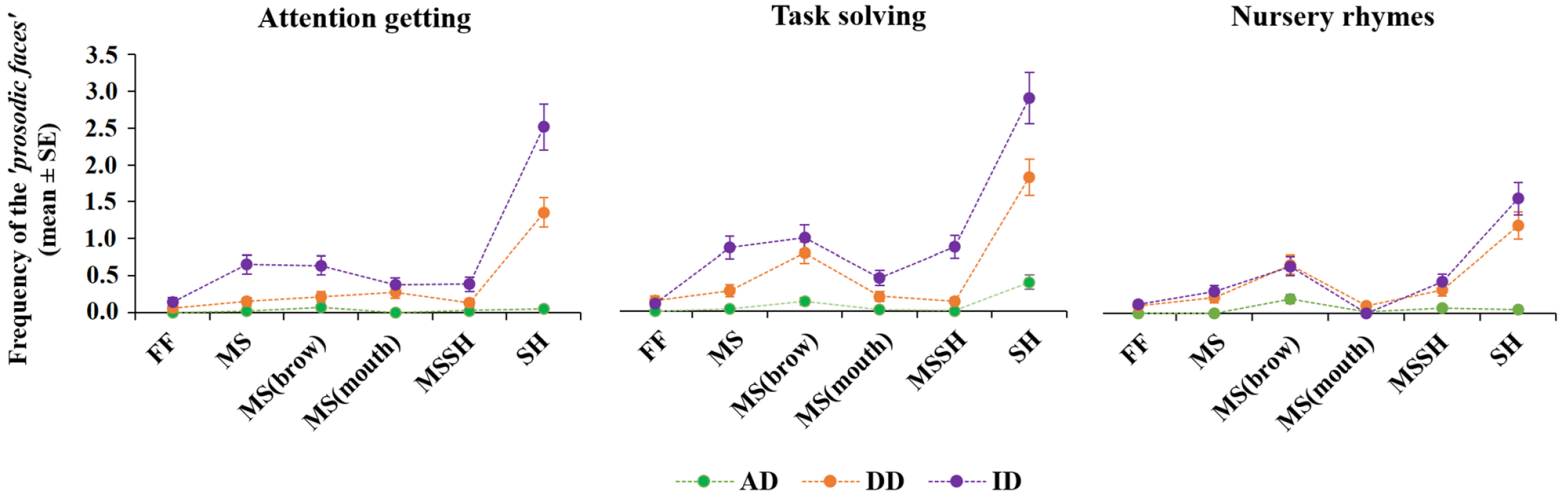
Mean (+ SE) frequencies of the *‘prosodic faces’* toward the three partners and situations. *AD* adult-directed, *DD* dog-directed, *ID* infant-directed. *FF*  *fish face*, *MS*  *mock surprise*, *MSSH* *mock surprise* + *special happy*, *SH* *special happy*.

We found that almost all of the prosodic face types were more frequently expressed during ID and DD as opposed to AD communication during the ‘Task solving’ situation (pairwise comparisons p < 0.05, Table S1a, Fig. 2). The only exception was the Fish Face (FF) which showed only marginal significance between ID and AD communication during this situation (p = 0.05, for details see Table S1a, Fig. 2). During ‘Attention getting’ situation, all face types were more frequent during ID than in AD communication, while only *MS, MS(mouth)* and *SH* faces were more frequent in DD as opposed to AD communication (all pairwise p < 0.05) and *MS(brow)* showed marginal significance (p = 0.05, Table S1a, Fig. 2). In comparison to AD communication, during ‘Nursery rhymes’ situation, the *MS, MS(brow), MSSH* and *SH* faces were more frequent during ID and DD communication (all pairwise p < 0.05, Table S1a, Fig. 2).

Results also showed that majority of the *‘prosodic faces’* were more frequent toward infants than toward dogs during the free speech situations (i.e. ‘Attention getting’ and ‘Task solving’: *MS, MSSH* and *SH;* all pairwise p < 0.05, in ‘Attention getting’ *MS(brow)* p = 0.005, in ‘Task solving’ *MS(mouth)* 0.028; Table S1a, Fig. 2). At the same time, no such difference has been found between DD and ID communication during the fixed speech situation (i.e. ‘Nursery rhymes’, all pairwise p > 0.05, Table S1a, Fig. 2).

Moreover, frequency analysis revealed a significant difference between face types, as *Special Happy (SH)* proved to be the most frequently displayed facial expression in ID and DD communication during the free speech situations (i.e. all pairwise p ≤ 0.001 in ‘Attention getting’ and ‘Task solving’; Table S1b, Fig. 2). *MS(brow)* proved to be the second most frequent face type and it was displayed more commonly than *FF* or *MS(mouth)* in DD and ID communication during ‘Task solving’ and ‘Nursery rhymes’ situations (all pairwise p < 0.02, Table S1b). Interestingly, we found that *Fish Face (FF)* was rarely displayed irrespectively of the partner and situation (for exact p-values see Table S1b, Fig. 2).

Our results showed that situation had an effect on the facial prosodic features of ID, DD and AD communication. Speakers displayed *SH* expression in AD communication, *MS(brow)* expression in DD- and *MSSH* expression in ID communication more during ‘Task solving’ situation compared to ‘Attention getting’ (all pairwise p < 0.02, Table S1c, Fig. 2). Similarly, *SH* toward all three partners and *MS* and *MSSH* expression in ID communication have been displayed more frequently during ‘Task solving’ situation compared to the ‘Nursery rhymes’ (all pairwise p < 0.05, Table S1c, Fig. 2). Interestingly, the frequency of *MS(mouth)* showed a different tendency in ID communication, as it was more common during ‘Nursery rhymes’ as opposed to ‘Task solving’ (p < 0.001, Table S1c, Fig. 2). Comparison of frequencies between ‘Attention getting’ and ‘Nursery rhymes’ situations showed a mixed picture. *MS* and *SH* expressions were more common in ID communication during ‘Attention getting’, while *MS(brow)* in DD and *MS(mouth)* in ID communication were more frequent during ‘Nursery rhymes’ (all pairwise p < 0.03, Table S1c, Fig. 2).

According to the Gender × Face Type interaction, pairwise comparisons revealed similar frequencies in all six *‘prosodic faces’* in female and male speakers (all pairwise p > 0.3, Table S2). Both genders displayed *SH* and *MS(brow)* expressions more frequently than the *FF* face type (all pairwise p < 0.01, Table S2, Figure S1). However, male speakers expressed the mouth and brow variation of *Mock Surprise* (*MS(brow)* and *MS(mouth)*) less frequently than the *Special Happy*, while no such difference could be found in female speakers (see Table S2, Figure S1).

### Intensity score analysis

We also examined the intensity of the movement of facial muscles (AUs) involved in the formation of the *‘prosodic faces’* during ID and DD communication. Models revealed several intensity differences between ID and DD facial prosody as well as between situations (see Supplementary Material 1.2 and Table S3 for full details).

Most common tendencies of the intensity results were the following:

1. ID and DD facial prosody differed in AU intensity in 10 out of the total of 25 models, and all 10 revealed greater intensity of the facial muscle movements toward infants than toward dogs (in all models F > 3.0, p < 0.05, see Table S3). Is important to note that in the most frequent SH face type, all relevant AUs were moving more intensively during ID, than in DD communication.
2. In 10 out of the 14 significant models on AU intensity, female and male speakers were displaying similarly intense facial movements. If gender of the speaker had an effect on AU intensity, it typically showed more intense facial movements in female than in male speakers. That is, more intense lip corner pulling (AU12) and lips part (AU25) movements in *Special Happy*, more intense eyebrow raise (AU1 + 2) in *MS(brow)*, and more intense mouth opening during ‘Task solving’ situation in *Fish Face* expression (all F > 7.8, p ≤ 0.006, Table S3). One exception was the eyelid movements (AU5/44) during *Special Happy* expression, which was more intense in male than in female speakers (F_1,495_ = 4.88, p = 0.028).
3. Situation had varying effects on AU intensity and these effects often manifested themselves through an interaction with the type of the partner and/or speaker’s gender (see Table S3). In five out of seven models that revealed significant effect of situation, pairwise comparisons showed more intense AU movements during ‘Task solving’ and/or ‘Nursery rhymes’ as opposed to ‘Attention getting’ (in all 5 models p < 0.05, Table S3).

**Figure 3:**
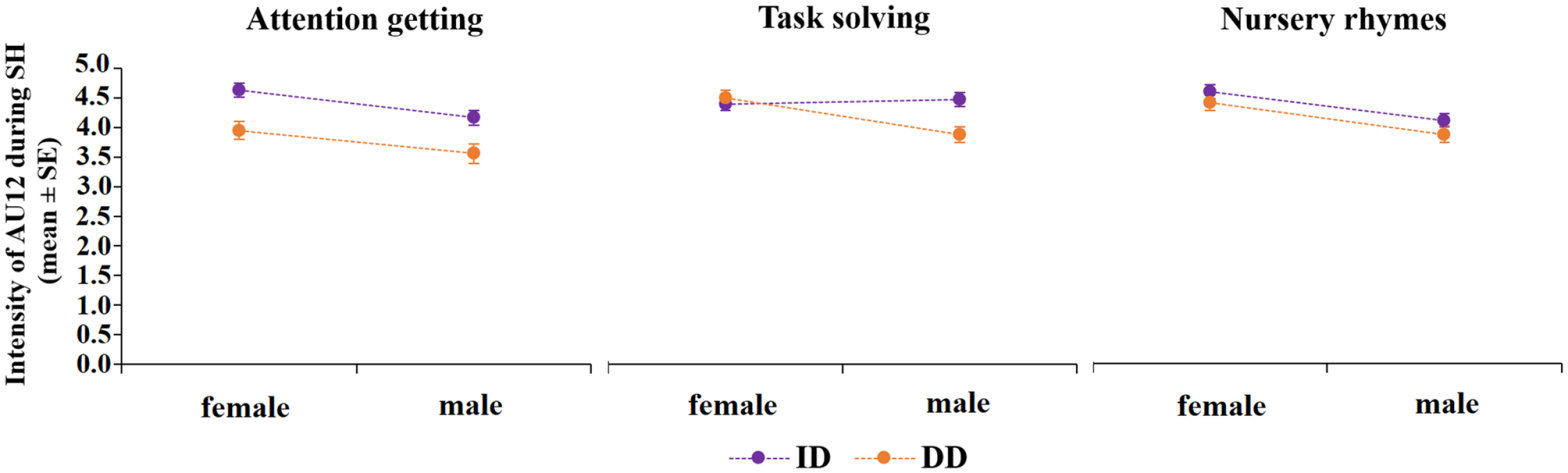
Mean (+ SE) scores of intensity of the lip corner puller (AU12) of male and female speaker’s *Special Happy (SH)* face in the three experimental situations. *DD* dog-directed, *ID* infant-directed speech.

Figure 3 demonstrates an example of the results of the intensity analyses: the effects of gender and partner on the intensity of lip corner pulling muscle (AU12, a typical component of *Special Happy* expression) in the different task situations (for more details see Supplementary Material 1.2 and Table S3).

## Discussion

The aim of the present study was to provide an AU-based description and comparative analysis of maternal and paternal facial prosodic features that accompany infant- dog- and adult-directed communication. Occurrence (i.e. frequency) and intensity of facial prosody were examined during natural face-to-face interactions and analysed in relation to the given situation and the speaker’s gender.

Our study provides evidence for the first time that infant-directed (ID) and dog-directed (DD) communication can be characterized by similar specific facial prosodic expressions of female and male speakers. In addition to the previously described three ‘prosodic faces’ toward infants^15,17^, here we have identified three novel expressions. Our results indicate that all six facial prosodic expressions are typical in both ID and DD communication but rare or completely missing form adult-directed (AD) communication. Importantly, we did not find any ID specific facial expression that was missing from DD prosody, note however, that the six *‘prosodic faces’* were less frequently and less intensively expressed by both male and female speakers toward dogs than towards infants. These results have important implications regarding the potential functions of ID facial prosody.

First, it is reasonable to assume that the *‘prosodic faces’* are expressed for multiple purposes that are similarly important when communicating with a preverbal infant and a pet dog. Based on previous studies on the functional similarities and differences between infants’ and dogs’ cognitive and emotional abilities^33,34,43,45^, we may assume that the *‘prosodic faces’* have an important role in calling and maintaining the partner’s attention, in increasing social bond between the partners and in fostering emotionally positive interactions. In line with previous findings on acoustic prosody these results further support the notion that people tend to use similar cues to engage infants and dogs in communicative interactions and this assumption can be extended to the facial features of prosody^24,25^.

At the same time, we can assume that the significantly less intense and frequent facial prosody toward dogs than toward infants might due to functions that are important when interacting with an infant, but less relevant when interacting with a dog (i.e. language tutoring, expressing intentions and in facilitating emotional synchrony between the speaker and the partner). Note, however, that our results are not conclusive, thus further studies (comparative analyses of the speaker’s and the partner’s facial expressions in ID and DD communicative changes during language tutoring and intention expressing situations) are needed to verify these assumptions about emotional synchrony and mirroring events.

Our results also indicate that one ID- and DD-specific facial expression, the *Special Happy*, significantly outweighs the other five ones in terms of frequency. This facial display dominated both ID and DD communicative exchanges regardless of the given situation, while there were faces, like the *Fish Face* and the *Mock Surprise mouth* variation that were rarely expressed. Huge variation in the number of occurrences suggests that distinct facial displays might have different roles and they contribute to the aforementioned functions of prosody to varying degrees. The *Special Happy* expression is basically an intense Duchenne smile that is typically accompanied with opened mouth^15,17^. The Duchenne smile is described as a spontaneous, genuine expression of an intense happy emotion that communicate strong social affiliation and interest to be engaged in social interaction^18^. Accompanied with opened mouth, the Duchenne smile is perceived even more happy as opposed to the closed mouth version of the same smile^46^. These indicate that female and male speakers of the present study intended to communicate their intense and honest happy emotion and their willingness to engage in social interaction in all task situations in which they interacted with their own preverbal infants or family dogs. The preference to look at positive facial expressions of humans has been shown in dogs and infants^32,33^, moreover, infants are more attentive toward smiles when accompanied by open-mouth (e.g.^47^). There is no doubt that such a smile has the potential to enhance positive interactions with an infant^7^, and now we can extend this assumption to canine companions.

In contrast to our prior hypothesis, the frequency of the *‘prosodic faces’* was not influenced by the interaction of the speaker’s gender and the given situation^19^. At the same time, intensity analysis of the most frequent face type, the *Special Happy* showed such interaction effect during ID and DD communication. That is, male speakers, displayed a more intense cheek raise (Action Unit 6), an important component of the Duchenne smile^18^, during the object-play situation (i.e. ‘Task solving’) in comparison to the fixed speech situation (i.e. ‘Nursery rhymes’). This is not true for female speakers: facial movements that are related to the Duchenne smile (i.e. Action Unit 6 & 12) were not influenced by the given situation. Note, however, that several Action Units showed more intense muscle movements in female than male speakers irrespective of the situation, including a more exaggerated lip corner pulling and mouth opening during *Special Happy* expression. Male speakers tended to open their eyes more than females, however, this difference was strongly dependent on the situation and the type of specific facial expression. These are in contradiction to previous findings on paternal and maternal smile types and emotional intensity of facial expressions toward infants^19,20^. This may be due to methodological differences between the present and previous studies, namely the age of the speakers, the age of the infant partners, differences in the experimental situations, and the measured variables, etc. In addition, cultural differences between American, Greek, and Hungarian parents can also have a significant effect on facial prosody (e.g.^15,48,49^), which raise interesting questions for future studies. In sum, the majority of our results, support the notion that facial displays accompanying infant-directed speech are rather universal among mothers and fathers (see^50^): paternal and maternal ID facial prosody share key features, and these similarities still exist in human–dog communicative exchanges.

In line with our hypothesis, we found several differences between free speech versus fixed speech situations. For example, during the fixed speech situation (i.e. ‘Nursery rhymes’) frequency of all *‘prosodic faces’* was similar during ID and DD communication, while several differences were found during the free speech situations (‘Task solving’ and ‘Attention getting’) in this regard. ‘Task solving’ situation evoked the most intense and frequent facial movements, including the most common *Special Happy* expression and this was so irrespective of the partner (i.e. infant/adult/dog). This finding is in line with our previous study on acoustic features of ID and DD prosody in which we observed the highest pitch^25^ in a very similar ‘Task solving’ situation. Speakers in this situation were asked to hide a toy and encouraged their partners (infant, dog, or adult) to find it. Given that praise occurred typically in this ‘Task solving’ situation (when the partner was successful in finding the hidden toy) it is reasonable to assume a functional link between the *‘prosodic faces’* and praise. This further confirms Chong et al..’s^15^ notion that the *Special happy* facial expression of mothers toward their infants is associated with praise.

Attention getting is usually listed among the main functions of prosody (e.g.^6^). Surprisingly, however, we found a lower rate of occurrence of the *‘prosodic faces’* in the ‘Attention getting’ situation than in the ‘Task solving’. Speakers in this situation were asked to attract their partners’ attention toward an object (i.e. a toy) as much as possible. Intense emotional facial expressions, however, can primarily serve to direct partners’ attention toward the face of the speaker (e.g.^51^). To effectively keep partners focused on the object, speakers thus may reduce their facial movements in the ‘Attention getting’ situation. However, more studies (comparisons of attention getting situations with and without the involvement of objects) are needed to confirm this conclusion.

One key finding of the current study is the identification and detailed characterization of three novel ID and DD specific facial expressions, in addition to the already published three faces by Chong and co-workers^15^. It is important to note that all newly identified *‘prosodic faces’* are variations and combinations of the previously described ones^15^ but they were reliably distinguishable from them. The reason why earlier studies described only three types of facial expressions may stem from methodological differences in the studies. Namely, Chong and co-workers^15^ measured the facial movements of mothers during one storytelling situation in which stories were carefully chosen to elicit a variety of emotions and related facial expressions (e.g., worried, happy) from the mother. In contrast, in the present study, we conducted three different situations that mimic everyday situations between the caregiver and the child. In two out of the three situations, speakers were allowed to talk freely, therefore the emotional content of their speech was not directly controlled by the experimental procedure. As facial expressions are highly influenced by the speaker’s intentions and emotions (e.g.^52^) it is possible that the emotionally less controlled situations evoked these novel and distinct faces. An alternative explanation would be that Chong et al.^15^ also observed these facial expressions, but classified them all as *Mock Surprise* or *Special Happy* varying only in intensity. For instance, it is possible that *Mock Surprise brow* facial expression was considered as the slight variation of *Mock Surprise* that lacks a jaw drop but includes intense brow raising movement and thus is not worthy of a separate rating. However, Chong et al.^15^ did not report a detailed analysis of AU intensity for the different facial expressions, so this possibility remains speculative. It should also be noted that differences in the infants’ age between the Chong et al.’s^15^ and our study might serve as another plausible explanation for the aforementioned differences. In the present study, infants’ mean age was 10 months (± 4.1 months), while in Chong et al. they were between 4 and 7 months^15^. As caregivers adjust their ID prosody to their infant’s capabilities and needs^53^, it is possible that these novel faces (i.e. *Mock surprise brow* and *mouth* variation as well as the *Mock Surprise* + *Special Happy* expression) are typically displayed when interacting with older, but not younger infants. Further studies are needed to investigate these possibilities.

In sum, the main findings of the current work can be summarized in the following way: (i) We provided evidence for the first time, that caregivers tend to use similar facial expressions when interacting with their preverbal infants and family dogs. (ii) Both female and male speakers used six distinct facial expressions, three were identified by the current study, that was typical when speaking to infants and dogs, but were rare or completely missing when speaking to adults. (iii) Facial prosody toward infants, dogs, and adults had context specific features, and situations that contain praise had the potential to evoke frequent and intense facial prosody. (iv) Our results highlighted the importance of infant-directed facial prosody in getting infant attention, conveying positive emotions through the interaction, and in strengthening the social bond between caregivers and infants. These results support the idea that the comparative investigation of facial prosody in human–dog and adult-infant communicative exchanges has the potential to broaden our knowledge about the significance of visual prosody and raises important questions for future research.

### Limitations of the study

This comparative study can be considered as the first step toward uncovering the role facial prosody plays in adult-infant and human–dog social interactions. Therefore, it has several limitations which should be investigated in future studies. (i) We studied ID, DD, and AD facial prosody during three situations that were evoking mostly positive emotions. We therefore could provide no information about visual prosody accompanied by negative emotions (e.g. fear, sadness, pain, etc.). (ii) Partner’s behaviour (i.e. facial expressions, attention, etc.) was not recorded in the present study, therefore we did not provide any information about the potential mirroring event or emotion synchronization. (iii) We did not analyse acoustic prosody in the present study, thus dynamics of the two modules (acoustic and facial prosody) were not studied. (iv) It has been raised that a friendly but unfamiliar adult is not a feasible control for infant-directed prosody because the lack of attachment between the speaker and the adult partner^54^. Here we used an unfamiliar friendly adult as a partner during the adult-directed communication in order to produce comparable data with relevant former studies (e.g.^15,19,25^). (v) Last but not least, potential underlying factors, like mental state (including depression), personality, experience with infants and dogs etc. of the speakers, that can have an effect on facial prosody were not analysed either. Future comparative studies are needed to study the aforementioned questions, for which, our study provided several important results and a feasible comparative framework.

## Materials and methods

### Ethic statement

This research was approved by the Human Research Ethics Committee (EPKEB) at Hungarian Academy of Sciences (No. 2015/23, 2022-85). In accordance with ethics approval, all parents completed an informed written consent to participate in the study and all methods were performed in accordance with the relevant guidelines and regulations of the EPKEB and the current laws of Hungary.

### Subjects

Sample size of the current study was based on a power analysis considering the design and statistical tests to be used (G*Power 3.1.9.7.). As there is no previous work that could provide specific effect size estimates, the a priori power analysis assumed a 'medium' effect size, α = 0.05 and power (1 − β) = 0.8. 42 participants from 22 families (N = 22 females; N = 20 males, age range: 18–45 years—for demographic details see Supplement Material 2.1 and Table S4) were recruited on a voluntary basis who had both a 1–18 months old child (13 girls, 9 boys, mean age 10.1 months ± 4.1 months SD) and an adult (over 1 year old) pet dog at home (16 females, 13 males, mean age ± SD 4.9 years ± 2.9 years).

All participants spoke Hungarian as their native language and from all participant, informed consent has been obtained to publish the information/image(s) in an online open-access publication.

### Apparatus

For video recordings we used a smartphone (Samsung Galaxy A70) on a tripod set to capture the participant’s face. We used three separate toy balls in the ID (sponge ball with a smiling face), DD (rubber dog toy) and AD (plastic ball) interactions, same in size and colour, for the ‘Attention getting’ and ‘Task solving’ situations (for details see below). For the ‘Nursery rhymes’ situation participants were allowed to use the printed version of the rhymes if needed.

### Procedure

Data acquisition took place in the participants’ home. During the recordings a female experimenter (the 1^st^ author of this paper) was present: she explained the tasks for the participants, controlled the starting and stopping of recordings and played the role of the partner during AD communication. Each participant was recorded speaking separately to their (i) infant (ID), (ii) dog (DD) and (iii) an adult experimenter (AD) in a within-subjects design. Video recordings of the participants were collected in two free speech situations (‘Attention getting’ and ‘Task solving’) in which they were encouraged to interact and speak freely without restrictions, and one fixed speech situation where they were asked to tell two nursery rhymes to the partner (‘Nursery rhymes’). Each recording lasted 30 to 40 s/situation (average length was 117  ± 11 s) and participants were encouraged to interact with all partners in a natural manner. Length of the recordings did not differ between ID, DD and AD communication (Linear GLMM, F_2,123_ = 0.744, p = 0.477). All three situations were recorded toward all three partners (ID, DD and AD). The order of ID, DD and AD communication were counterbalanced across participants.

‘Attention getting’: participants were instructed to call the partner’s (infant, dog or adult) attention toward a small red ball, and they were asked to use the following words as frequently as possible: “red”, “ball”, “rolling” (in Hungarian these words are “piros”, “labda”, “gurul”). We asked this because these words in Hungarian contain all three vowels important for examining hyperarticulation (i, a, u,—see also^25^). We used three different balls for ID, AD and DD communication due to hygienic reasons (for details see Apparatus).

‘Task solving’: participants were instructed to play a hide-and-seek game with the partner by using the same red ball as in the ‘Attention getting’ situation. They were asked to hide the ball into one of their hands then ask the partner to find it. They were also instructed to use the words “red”, “ball”, “rolling” as frequently as possible during this phase too.

‘Nursery rhymes’: participants were asked to pick two rhymes they already know out of 8 and tell these rhymes to the partner.

### Data analysis

As the first step, one of the experimenter (the 2nd author of this paper, Coder 1) watched all of the recordings (42 speakers × 3 partners × 3 situations = 378 videos in total) while aiming to identify all facial movements that differed from a neutral facial expression, including the three faces described by Stern and Chong et al.^15,17^. She, then saved each of these facial expressions as individual pictures which resulted in a total of 1084 pictures. It is important to note that we did not analyse duration of the facial expressions in the present paper. Some expressions, *Special Happy* for instance (see details below), are typically presented for long durations with varying intensity, but without changing into neutral or into other face types. In this case, it was included to our sample as one *Special Happy* expression, and the screen shot was taken at the most intense moment (as judged by Coder 1). As a next step, Coder 1 classified and sorted the pictures which were fitting into the three categories according to Stern and Chong et al.^15,17^:

### Fish face (FF)

The name of this facial expression stems from the typical position which the crinkled lips and the slightly open mouth create. When examining the face’s partition by Ekman et al.^55^ the changes in the action unit (AU) 18 and 22 can be associated with this type of ID expression.

### Mock surprise (MS)

This term sums up the simultaneous appearance of the raised eyebrows and wide-open eyes and mouth with a lip corner pull (i.e. AU 12).

### Special happy (SH)

This particular expression is more than a casual smile because without exception the mouth is at least slightly open. Similar to the Duchenne smile, its most important features are the cheek raise (corresponding to AU 6) and lip corner pull (AU 12). According to Ekman et al.^55^, AU 12—i.e. facial changes produced by the zygomatic major muscles—are always an indicator of a positive emotional state. For differentiating it from a simple, but bright smile, the fine wrinkles around the eyes’ outer side were taken into account.

Note, however, that 35.6% of the pictures did not fit neatly into any predetermined category. The experimenter therefore sorted these pictures into three additional categories on the basis of changes in facial muscle movements (*Mock Surprise brow; Mock Surprise mouth; Mock Surprise* + *Special Happy—*for a detailed description of these faces see Results)*.* Inter-observer reliability scores were measured between two additional independent observers (the 1^st^ and 3^rd^ authors of this paper, Coder 2 and 3) for the observed facial expressions. Percentage agreements and Cohen’s kappa coefficients were calculated for each category of facial expressions: % agreement ranged from 95.04 to 99.01 and Cohen’s kappa values were between 0.84 and 0.91 showing a high level of reliability (Table 2). Note, however, that in case of disagreement between the three coders (Coder 1, 2 and 3), the categorization of the observed facial expression was based on majority opinion.

**Table 2:** Percentage agreements and Cohen’s kappa coefficients for the six facial expression types.

	SH	FF	MS	MSSH	MS (brow)	MS (mouth)
N (1084 in total)	547	33	118	110	203	73
%	98.72	99.01	96.61	98.19	95.42	98.81
Cohen’s κ	0.84	0.86	0.84	0.91	0.86	0.91

**Figure 4:**
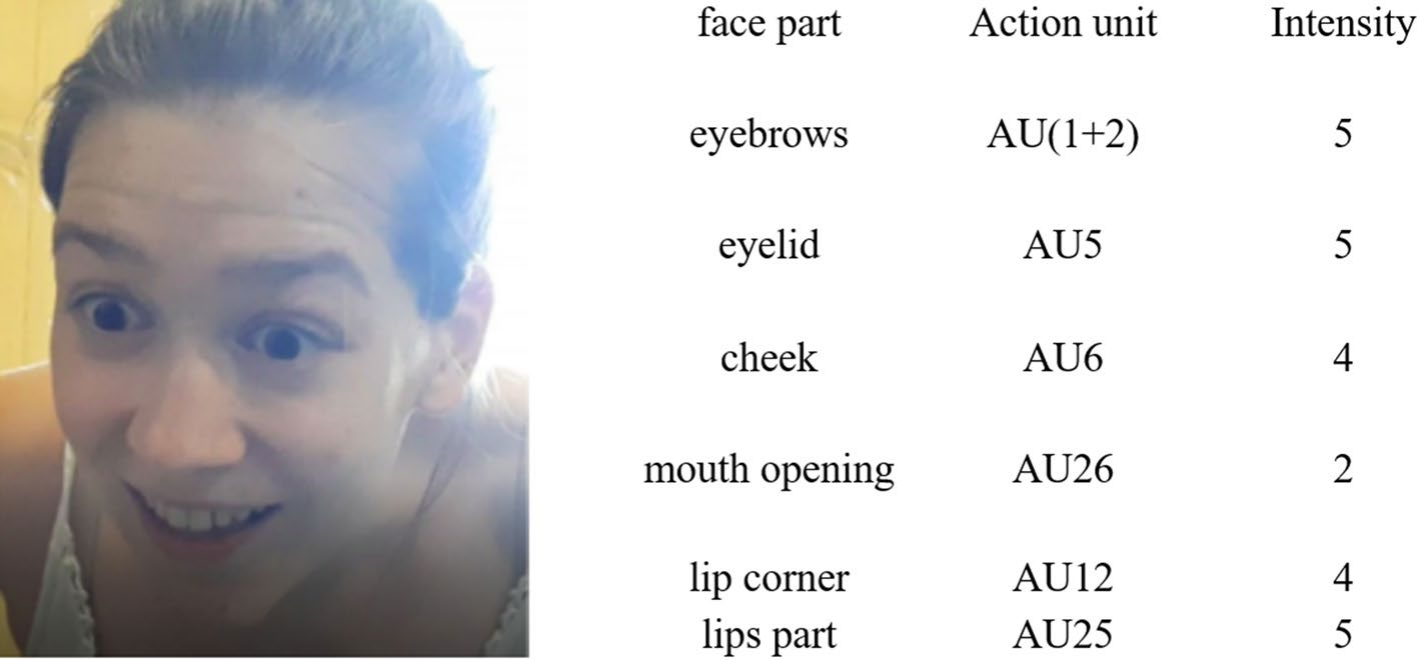
A typical *MSSH* facial expression and the corresponding FACS coding.

As a second step, Coder 2 and 3 also analysed the pictures using the golden-standard Facial Action Coding System (FACS^56^). This method defines the intensity of the given facial expressions by assigning values on a unique scale to specific areas of the face referred to as Action Units (AUs). 28 pictures have been excluded form FACS analysis because of the angle of the face or the light conditions made reliable coding impossible. This resulted a total of 1056 pictures for FACS coding. For detailed description of the analysed AUs see Supplementary Material (Table S5). In accordance with the instructions and criteria for calculating intensity levels illustrated in the *Manual for Facial Action Coding System*^57^ Coder 2 and 3 assigned the corresponding intensity score to all movement types (0: *neutral/absent*; 1: *trace,* 2: *slight;* 3: *marked/pronounced;* 4: *severe;* 5: *maximum*). In case of the mouth opening and eyelid movements we used a slightly different scale (for details see Supplementary Material). Figure 4 shows one example of typical infant-directed facial expressions and corresponding FACS coding.

Inter-observer reliability scores were measured between two additional independent observers (Coder 2 and 3) for the FACS coding.

Coder 2 (AG) coded 3 out of the 6 facial expression types (*SH, MSSH* and *FF*), while Coder 3 (LAF) coded the remaining 3 types (*MS, MS(brow)* and *MS(mouth)*). Inter-observer agreements for FACS coding were assessed by means of parallel coding of the 20% of the total sample. The integrated intra-class correlation coefficient (ICC) was 0.84, which is considered to be good^58^.

### Statistical analysis

The effects of the partner (AD, DD & ID), situation (‘Attention getting’, ‘Task solving’, ‘Nursery rhymes’), the speakers’ gender (female/male) and their interactions on both the frequency and intensity of facial expressions were analysed by using Generalized Linear Mixed Models (GLMMs). Backward elimination technique was applied in all models in a stepwise manner for removing the non-significant interactions and main effects from the model. Identification number for each participants was included as a random grouping factor in order to control for repeated measurement. In all post-hoc comparisons Bonferroni correction was applied. For all statistical analysis we used IBM SPSS Statistics (version 23) software.

Frequency analyses were carried out by using GLMM with Negative Binomial regression. Fixed factors were Gender (female/male); Partner (AD, DD, ID); Situation (‘Attention getting’, ‘Task solving’, ‘Nursery rhymes’), and Face Type (*SH, MSSH, MS, MS*(*brow*),* MS*(*mouth*),* FF*). All two- and three-way interactions were also included in the model (Gender × Partner; Gender × Face Type; Gender × Situation; Situation × Face Type; Situation × Partner, Partner × Face Type; Gender × Partner × Situation, Gender × Partner × Face Type, Gender × Situation × Face Type, Partner × Situation × Face Type).

For intensity analyses we used separate GLMMs for Gaussian distribution for each relevant AU in each face type (see Table S5 for the list of analysed AUs). We considered an AU relevant if it was activated (i.e. intensity score differed from 0) in that specific face type in at least 50% of cases (for details see Table 1).

Since all face types were markedly underrepresented in AD communication (only 48 out of 1084 faces) and the *FF* expression was completely missing, we decided to compare intensity between DD and ID communication only. In all models fixed factors were Gender (female, male); Partner (DD, ID) and Situation (‘Attention getting’, ‘Task solving’, ‘Nursery rhymes’). All two- and three-way interactions were also examined (Partner × Situation; Partner × Gender; Gender × Situation; Gender × Partner × Situation).

## Supplementary Information

Supplementary Information.

Supplementary Information.

## Data Availability

The data analysed during the current study are available from the corresponding author on reasonable request. Raw data: frequency and intensity data can be found in Supplementary Dataset 1.
