# Supplementary material for: Six facial prosodic expressions caregivers similarly display to infants and dogs
Source: Sci Rep. 2023 Jan 17;13:929. doi: 10.1038/s41598-022-26981-7 (PMC9845226; doi:10.1038/s41598-022-26981-7)
Supplement: Supplementary file 1 — Supplementary Information. [file 41598_2022_26981_MOESM1_ESM.pdf]

Supplementary Material

Six facial prosodic expressions caregivers similarly display to infants and dogs

Anna Gergely, Édua Koós-Hutás, Lőrinc András Filep, Anna Kis, József Topál

1. Results

1.1.Frequency of the *'prosodic faces'*

Non-significant interaction terms and a main effect were removed from the model in the following order: Gender × Condition × Face Type, Gender × Condition × Situation, Gender × Situation × Face Type; Gender × Condition, Gender × Situation, Gender × Face Type, Situation × Face Type. The final model revealed significant Condition × Situation × Face Type ($F_{34,2208}$=1.78, p=0.004) three-way (Table S1a,b,c) and Condition × Face Type ($F_{10,2208}$=2.11, p=0.021, not relevant because it is included in the three-way interaction), Gender × Face Type ($F_{5,2208}$=3.48, p=0.006) two-way interactions (Table S2, Figure S1).

Table S1a. Summary of pairwise comparisons revealed by the Negative Binomial GLMM on the frequency data during the Condition × Situation × Face Type three-way interaction. < = less frequent (p<0.05), ns = p>0.05

	most common relations	FF	MS	MS(brow)	MS(mouth)	MSSH	SH
'Attention getting'	AD < DD	ns	0.037	*0.05*	0.001	ns	<0.001
	AD < ID	0.032	<0.001	<0.001	<0.001	0.001	<0.001
	DD < ID	ns	<0.001	0.005	ns	0.033	<0.001
'Task solving'	AD < DD	0.032	0.002	<0.001	0.018	0.017	<0.001
	AD < ID	*0.05*	<0.001	<0.001	<0.001	<0.001	<0.001
	DD < ID	ns	0.001	ns	0.027	<0.001	0.001
'Nursery rhymes'	AD < DD	ns	0.005	0.003	ns	0.012	<0.001
	AD < ID	ns	0.001	0.003	ns	0.002	<0.001
	DD < ID	ns	ns	ns	ns	ns	ns

Table S1b. Summary of pairwise comparisons revealed by the Negative Binomial GLMM on the frequency data during the Condition × Situation × Face Type three-way interaction. underlined= opposite to trend, < = less frequent (p<0.05), > = more frequent (p<0.05), ns = p>0.05

	most common relations	AD	DD	ID
'Attention getting'	SH > FF	ns	<0.001	<0.001
	SH > MS	ns	<0.001	<0.001

	SH > MS(brow)	ns	<0.001	<0.001
	SH > MS(mouth)	ns	<0.001	<0.001
	SH > MSSH	ns	<0.001	<0.001
	MS(brow) > MS(mouth)	ns	ns	ns
	MS(brow) > MS	ns	ns	ns
	MS(brow) > MSSH	ns	ns	ns
	MSSH > MS(mouth)	ns	ns	ns
	MSSH vs MS	ns	ns	ns
	MS > MS(mouth)	ns	ns	ns
	FF < MS	ns	ns	0.002
	FF < MS(brow)	ns	ns	0.002
	FF < MS(mouth)	ns	ns	ns
	FF < MSSH	ns	ns	ns
'Task solving'	SH > FF	0.001	<0.001	<0.001
	SH > MS	0.002	<0.001	<0.001
	SH > MS(brow)	ns	0.001	<0.001
	SH > MS(mouth)	0.002	<0.001	<0.001
	SH > MSSH	0.001	<0.001	<0.001
	MS(brow) > MS(mouth)	ns	0.001	0.016
	MS(brow) > MS	ns	0.008	ns
	MS(brow) > MSSH	ns	<0.001	ns
	MSSH > MS(mouth)	ns	ns	ns
	MSSH vs MS	ns	ns	ns
	MS > MS(mouth)	ns	ns	ns
	FF < MS	ns	ns	<0.001
	FF < MS(brow)	ns	<0.001	<0.001
	FF < MS(mouth)	ns	ns	0.012
	FF < MSSH	ns	ns	<0.001
'Nursery rhymes'	SH > FF	ns	<0.001	<0.001
	SH > MS	ns	<0.001	<0.001

	SH > MS(brow)	ns	ns	0.001
	SH > MS(mouth)	ns	<0.001	<0.001
	SH > MSSH	ns	<0.001	<0.001
	MS(brow) > MS(mouth)	ns	<0.001	<0.001
	MS(brow) > MS	ns	0.014	ns
	MS(brow) > MSSH	ns	ns	ns
	MSSH > MS(mouth)	ns	ns	<0.001
	MSSH vs MS	ns	ns	ns
	MS > MS(mouth)	ns	ns	0.003
	FF < MS	ns	ns	ns
	FF < MS(brow)	ns	<0.001	0.001
	FF < MS(mouth)	ns	ns	ns
	FF < MSSH	ns	0.044	0.038

Table S1c. Summary of pairwise comparisons revealed by the Negative Binomial GLMM on the frequency data during the Condition × Situation × Face Type three-way interaction. underlined= opposite to trend, < = less frequent (p<0.05), > = more frequent (p<0.05), ns=p≥0.05

	most common relations	FF	MS	MS(brow)	MS(mouth)	MSSH	SH
AD	AG < TS	ns	ns	ns	ns	ns	0.002
	AG vs. NR	ns	ns	ns	ns	ns	ns
	TS > NR	ns	ns	ns	ns	ns	0.002
DD	AG < TS	ns	ns	0.001	ns	ns	ns
	AG vs. NR	ns	ns	AG<NR 0.003	ns	ns	ns
	TS > NR	ns	ns	ns	ns	ns	0.049
ID	AG < TS	ns	ns	ns	ns	0.012	ns
	AG vs. NR	ns	AG>NR 0.027	ns	AG<NR <0.001	ns	AG>NR 0.003
	TS > NR	ns	0.002	ns	TS<NR <0.001	0.018	<0.001

Table S2. Summary of pairwise comparisons revealed by the Negative Binomial GLMM on the frequency data during the Gender x Face Type two-way interaction. Pairwise comparisons that were non-significant in both female and male spekaers were missed out from the table (i.e. SH vs. MS, MS vs MSSH etc.). > = more frequent (p≤0.05), ns p>0.05

female vs. male speakers	p>0.3 in all six Face Type pairwise comparisons	
most common relations	women	men
SH > FF	p<0.001	p<0.001
SH > MS(mouth)	ns	p<0.001
SH > MS(brow)	ns	0.003
MS(brow) > FF	0.003	p<0.001

###Insert Figure S1 here

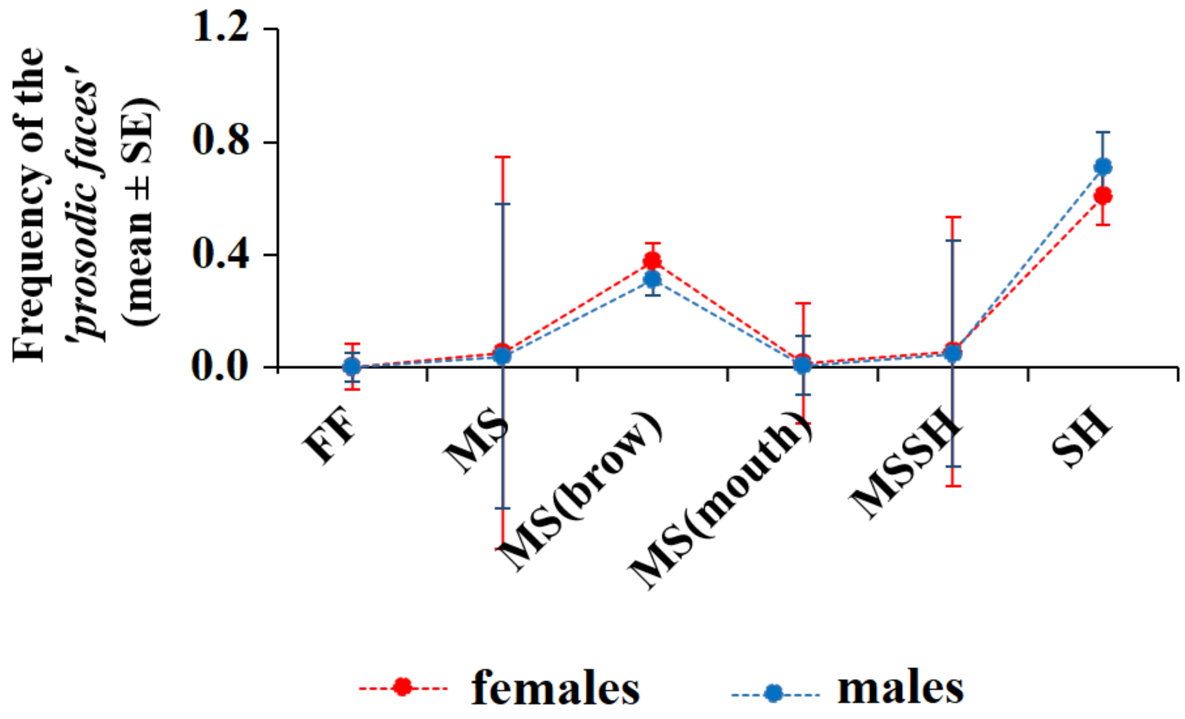

1.2.Intensity of the *‘prosodic faces’*

Intensity results of each relevant AU are presented separately from the most to the least frequent Face Type.

Table S3. Summary of the intensity results (Linear GLMM). AU= Action Unit, DD=dog directed, ID= infant directed, AG=’Attention getting’, NR=’Nursery rhymes’, TS=’Task solving’, ♀=female speakers, ♂=male speakers, > = more intense AU movement (p<0.05), ns=all main effects and interactions p>0.05), - = the AU was not relevant-not included to the model. bold= most frequent tendency, underlined= opposite to trend

movement (AU number)	Special Happy	Mock Surprise (brow)	Mock Surprise + Special Happy	Mock Surprise	Mock Surprise (mouth)	Fish Face
eyebrow raise (1+2)	**-**	♀>♂<br>$F_{1,160}$=7.84, p=0.006	ns	ns	-	**NR > AG**<br>$F_{2,29}$=3.53, p=0.042<br>(pairwise p=0.049)
eyelids (5/44)	**ID>DD** $F_{1,495}$=9.96, p=0.002<br><u>♂>♀</u> $F_{1,495}$=4.88, p=0.028<br><u>AG>TS</u>, NR $F_{2,495}$=6.26, p=0.002<br>(all pairwise p<0.04)	**ID>DD**<br>$F_{1,157}$=10.16, p=0.002	ns	**ID>DD**<br>$F_{1,108}$=9.23, p=0.003	-	**-**
cheek (6)	AG: **ID>DD** $F_{2,511}$=4.85, p=0.008<br>♂: **TS>AG**, NR $F_{2,511}$=3.17, p=0.043<br>(all pairwise p≤0.001)	-	AG: **ID>DD**<br>DD: **NR,TS > AG**<br>$F_{2,105}$=6.57, p=0.002<br>(all pairwise p≤0.003)	-	-	-
lip corner puller (12)	♀>♂ $F_{1,512}$=10.44, p=0.001<br><br>AG, TS: **ID>DD**<br>DD: **NR,TS > AG**<br>($F_{2,512}$=3.55, p=0.029, all pairwise p<0.03)	NR>TS<br>($F_{2,159}$=3.67, p=0.028, pairwise p=0.023)	AG, TS: **ID>DD**<br>DD: **NR > AG**<br>$F_{2,105}$=5.15, p=0.007<br>(all pairwise p≤0.005)	-	-	-
lip protruding (18)	-	-	-	-	-	ns
lips part (25)	DD, ID**:** ♀>♂<br>♂: **ID>DD**<br>($F_{1,507}$=11.97, p=0.001, all pairwise p<0.001)	ns	**ID>DD**<br>$F_{1,108}$=9.73, p=0.002	ns	ns	ns
mouth opening (26- 27)	-	ns	ns	ns	ns	**ID>DD**<br>$F_{1,22}$=9.24, p=0.006<br>TS: ♀>♂<br>$F_{2,22}$=4.13, p=0.03

2. Materials and Methods

2.1 Subjects

45.2% of the participants live in the capital city of Hungary while 33.3% of the participants' home is found in the agglomeration of Budapest. The most frequent educational attainment was university/college degree (69.1 %), 19% of the participants possess a high school diploma and the remaining 11.9% completed post-graduate studies. For demographic details see Table S4.

Table S4. Demographic details of the participants and their partners in infant-directed (ID) and dog-directed (DD) conditions. FIN= family identity number, PIN= participant identity number

FIN	PIN	speaker's gender	age	education	infant's age (months)	infant's gender	dog's age (years)	breed	dog's gender
1	1	female	26-35	post-graduate	8.9	female	2.5	bichon havanese	female
	2	male	26-35	univesity/ college degree					
2	3	female	26-35	univesity/ college degree	13.8	male	3.5	poodle	male
	4	male	36-45	univesity/ college degree					
3	5	female	36-45	univesity/ college degree	5.8	female	7.5	border collie	female
	6	male	36-45	univesity/ college degree			11.5	border collie	female
4	7	female	26-35	post-graduate	17.2	male	5.0	Irish setter	female
	8	male	26-35	univesity/ college degree					
5	9	female	26-35	univesity/ college degree	5.3	male	6.0	Australian shepherd	female
	10	male	26-35	univesity/ college degree					
6	11	female	26-35	univesity/ college degree	11.0	female	3.5	German pointer	male

7	12	female	26-35	secondary school-leaving exam	13.6	male	5.5	Dachshund	male
	13	male	26-35	univesity/ college degree					
8	14	female	36-45	univesity/ college degree	7.9	female	10.0	beagle	male
	15	male	36-45	secondary school-leaving exam					
9	16	female	36-45	univesity/ college degree	6.4	male	2.5	Weima-raner pointer	female
	17	male	26-35	univesity/ college degree			5.0	mixed	male
10	18	female	36-45	univesity/ college degree	12.2	female	2.0	Dachshund	female
	19	male	36-45	univesity/ college degree			3.5	kangal shepherd	female
11	20	female	26-35	secondary school-leaving exam	15.6	female	8.0	mixed	male
	21	male	26-35	secondary school-leaving exam			11.5	mixed	female
12	22	female	26-35	univesity/ college degree	10.5	male	2.0	German shepherd	female
13	23	female	26-35	univesity/ college degree	16.9	female	5.0	border collie	female
	24	male	36-45	post-graduate					
14	25	female	26-35	secondary school-leaving exam	13.3	male	5.0	mixed	female
	26	male	36-45	secondary school-leaving exam			8.0	mixed	male

15	27	female	26-35	univesity/ college degree	14.2	female	1.0	mudi	female
	28	male	26-35	univesity/ college degree					
16	29	female	26-35	univesity/ college degree	8.4	female	3.0	mixed	female
	30	male	26-35	univesity/ college degree					
17	31	female	26-35	university/ college degree	10.3	female	0.5	mixed	male
	32	male	26-35	university/ college degree					
18	33	female	26-35	secondary school-leaving exam	8.0	female	5.0	mixed	male
	34	male	36-45	univesity/ college degree			3.0	mixed	male
19	35	female	18-25	univesity/ college degree	3.0	female	5.0	mixed	male
	36	male	26-35	univesity/ college degree					
20	37	female	26-35	post-graduate	4.2	male	2.0	great dane	male
	38	male	26-35	univesity/ college degree					
21	39	female	26-35	univesity/ college degree	7.1	male	7.0	mixed	female
	40	male	26-35	univesity/ college degree					
22	41	female	36-45	post-graduate	7.7	female	6.0	mixed	male
	42	male	36-45	secondary school-leaving exam			3.5	mixed	female

2.2. Data analysis

Table S5. Relevant Action Units for the present study

Facial part	Motion/Movement type		Action Unit	
Upper Face	Eyebrow	raise	*Inner Brow Raiser*	AU 1
			Outer Brow Raiser	AU 2
Eyes	Eyelid	raise	*Upper Lid Raiser*	AU 5
		lowering	*Squint*	AU 44
Cheek	Cheek raise		*Cheek Raiser & Lid Compressor*	AU 6
Lower Face	Mouth opening		*Jaw Drop*	AU 26
Mouth			*Mouth Stretch*	AU 27
	Position of the lip corner		*Lip Corner Puller*	AU 12
	Rate of lip protruding		*Lip Pucker*	AU 18
	Distance between the lips		*Lips Part*	AU 25

In case of the mouth opening we used a different scale from the default one, since the movements marked by AU 26 and 27 can be considered as different parts of the same continuum: neutral/absent = **0**; AU26, intensity 1 = **0.5**; AU26, intensity 2 = **1**; AU26, intensity 3 = **1.5**; AU26, intensity 4 = **2**; AU26, intensity 5 = **2.5**; AU27, intensity 1 = **3**; AU27, intensity 2 = **3.5**; AU27, intensity 3 = **4**; AU27, intensity 4 = **4.5**; AU27, intensity 5 = **5**

Similarly, in eyelid movements, since AU 5 and AU 44 can be considered as each other's opposite, we used the following scale: AU44, intensity 5 = **-5**; AU44, intensity 4 = **-4**; AU44, intensity 3 = **-3**; AU44, intensity 2 = **-2**; AU44, intensity 1 = **-1**; neutral/absent = **0**; AU5, intensity 1 = **1**; AU5, intensity 2 = **2**; AU5, intensity 3 = **3**; AU5, intensity 4 = **4**; AU5, intensity 5 = **5**.

Figure legend

Figure S1. Frequency of the *'prosodic faces'* in female and male speakers.
