# Supplementary figures and images for: Six facial prosodic expressions caregivers similarly display to infants and dogs
Source: Sci Rep. 2023 Jan 17;13:929. doi: 10.1038/s41598-022-26981-7 (PMC9845226; doi:10.1038/s41598-022-26981-7)

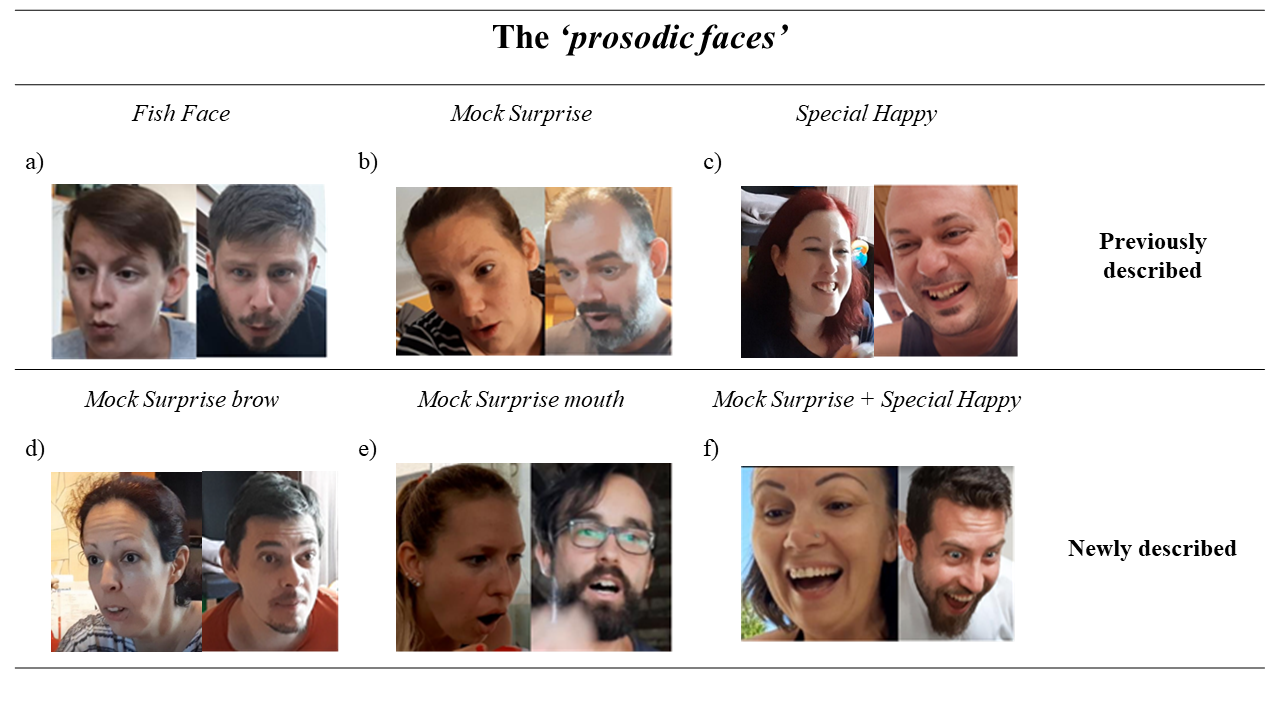

Supplement: Supplementary file 2 — Supplementary Information. [file 41598_2022_26981_MOESM2_ESM.tif]
